# Prevention of central line-associated bloodstream infections: a survey of ICU nurses’ knowledge and practice in China

**DOI:** 10.1186/s13756-020-00833-3

**Published:** 2020-11-16

**Authors:** Xiuwen Chi, Juan Guo, Xiaofeng Niu, Ru He, Lijuan Wu, Hong Xu

**Affiliations:** 1 School of Nursing, Guangdong Medical University, No. 1 Xincheng Road, Songshan Lake Science and Technology Industrial Park, Dongguan, 523808 Guangdong China; 2 School of Nursing, Heze Medical College, Heze, 274000 Shandong China; 3 Department of Upper Extremity Orthopedics, Eastern Hospital of the First Affiliated Hospital, Sun Yat-Sen University, Guangzhou, 510700 Guangdong China; 4 Department of Intensive Care Unit, Longgang Central District Hospital, Shenzhen, 518116 Guangdong China

**Keywords:** Central line-associated bloodstream infections, Evidence-based guidelines, ICU, Nurses

## Abstract

**Background:**

Central line-associated bloodstream infections (CLABSI) are largely preventable when evidence-based guidelines are followed. However, it is not clear how well these guidelines are followed in intensive care units (ICUs) in China. This study aimed to evaluate Chinese ICU nurses’ knowledge and practice of evidence-based guidelines for prevention of CLABSIs issued by the Centers for Disease Control and Prevention, US and the Department of Health UK.

**Method:**

Nurses completed online questionnaires regarding their knowledge and practice of evidence-based guidelines for the prevention of CLABSIs from June to July 2019. The questionnaire consisted of 11 questions, and a score of 1 was given for a correct answer (total score = 0–11).

**Results:**

A total of 835 ICU nurses from at least 104 hospitals completed the questionnaires, and 777 were from hospitals in Guangdong Province. The mean score of 11 questions related to evidence-based guidelines for preventing CLABSIs was 4.02. Individual total scores were significantly associated with sex, length of time as an ICU nurse, educational level, professional title, establishment, hospital grade, and incidence of CLABSIs at the participant’s ICU. Importantly, only 43% of nurses reported always using maximum barrier precautions, 14% of nurses reported never using 2% chlorhexidine gluconate for antisepsis at the insertion site, only 40% reported prompt removal of the catheter when it was no longer necessary, and 33% reported frequently and routinely changing catheters even if there was no suspicion of a CLABSI.

**Conclusion:**

Chinese ICU nurses in Guangdong Province lack of knowledge and practice of evidence-based guidelines for the prevention of CLABSIs. National health administrations should adopt policies to train ICU nurses to prevent CLABSIs.

## Background

Central line-associated bloodstream infections (CLABSIs) are the most common complication of central venous catheters (CVCs), with an incidence of 4.1 per 1000 central line days [1]. CLABSIs are associated with increased morbidity, mortality, and medical costs [2]. A meta-analysis shows that patients with CLABSI have an 2.75-time higher risk for hospital death than those without CLABSI [3]. It has been shown that CLABSI is associated with high-cost burden, accounting for approximately $46,000 per case [4]. CLABSIs are considered to be largely preventable when evidence-based guidelines for the insertion and maintenance of CVCs are followed [5], such as hand washing, using full-barrier precautions during central venous catheter insertion, cleaning the skin with chlorhexidine, and removing unnecessary catheters. Most intensive care units (ICUs) in developed countries now report CLABSI rates of zero or close to zero [6]. However, the overall incidence of CLABSIs in ICUs in China is about 2.81‰ [7].

It has been confirmed that evidence-based interventions can reduce the incidence of CLABSIs [8], including nursing bundles coupled with education and the commitment of both staff and institutions. The Keystone ICU Evidence-Based Intervention Program initiated by the Michigan Health and Hospital Association (MHA) reduced the median incidence of CLABSIs from 2.7/1000 catheter days at baseline to zero in the first 3 months [5]. Ullman et al. [9] reported that the knowledge and practice for preventing CVC-related infections varied greatly in pediatric ICU nurses in Australia and New Zealand, and many were inconsistent with guideline recommendations, such as maximum sterile barrier precautions, use of suture-less securement devices, replacing transparent dressing at least every 7 days. The authors concluded that an overall improvement of knowledge of evidence-based guidelines was needed. Bianco et al. [10] reported that evidence-based policies and training can help nurses increase their knowledge, practice, and attitude towards preventing CLABSIs. The authors also reported that despite new evidence, many non-evidence-based practices continue.

A number of researchers have designed questionnaires to investigate ICU nurses’ knowledge of guidelines to prevent CLABSIs [11–14]. Labeau et al. [8] developed and validated a questionnaire for evaluating critical care nurses' knowledge of evidence-based guidelines for preventing CVC infections. The questions in Labeau et al.’s questionnaire are very important and concise, and suitable for large-scale investigations. Studies performed in Poland, Australia, and other countries have referenced the questionnaire developed by Labeau et al. [9, 15].

The CLABSI rate in ICUs in China (2.81‰ [7]) is greater than in most developed countries [6]. The purpose of this study was to use questionnaires to determine Chinese ICU nurses’ knowledge of evidence-based guidelines for preventing CLABSIs issued by the Centers for Disease Control and Prevention, US [16] and the Department of Health UK [17], and their frequency of practicing guideline recommendations.

## Methods

### Study design and subjects

This was a cross-sectional descriptive survey study. It was conducted using an online tool, Questionnaire Star (https://www.wjx.cn/wjx/design/previewmobile.aspx?activity=41939767&s=1). The study was conducted by respondent driven sampling (RDS) from June to July 2019. The selection criteria for the respondents were: (1) nurses worked in the hospital comprehensive ICU, in which patients used central venous catheters; (2) the nurses participated in the maintenance of central venous catheters.

There were three different sources of respondents. (1) Nurses from 6 hospitals with comprehensive ICUs were directly invited to participate in the study and complete the online questionnaire since we know the head nurse of ICU of these six hospitals. (2) Students who graduated from Guangdong Medical University were asked to send out invitations to nurses to complete the questionnaire. (3) The information about the questionnaire was also distributed through the WeChat group of students who graduated from the Nursing College of Guangdong Medical University. Nurses were also asked to inform other ICU nurses about the study and ask them to complete the questionnaire. All respondents voluntarily participated in the survey, and the questionnaire was collected anonymously. Ethics approval for this study was obtained from the Ethics Committee, Affiliated Hospital of Guangdong Medical University, Zhanjiang, China (Reference number: YJYS2018075). Completion and return of the questionnaires was considered consent and voluntary participation in this study.

The study was aimed at ICU nurses in Guangdong Province. However, after it was found that a few nurses from other provinces were completing the questionnaire, we set a limitation such that only IP addresses locating in Guangdong Province could access the online questionnaire.

The nurses were grouped according to the amount of time they had been working as an ICU nurse (< 1, 1– < 3, 3– < 5, 5–10, > 10 years), as previously described [11].

### Questionnaires and data collection

The questions in the questionnaire of this study were mainly based on the guidelines issued by the Centers for Disease Control and Prevention (CDC), U.S [16]. and the Department of Health UK [17]. The questionnaire used in this study was titled ‘Knowledge of CLABSI Prevention Questionnaire’. It was based on the questionnaire developed by Labeau et al. [11], who authorized its modification and use in this study. The modifications were made based on several current literature references, and current evidence-based recommendations for the prevention of CLABSIs. The questionnaire consisted of 11 questions, and a score of 1 was given for a correct answer and a score of zero for a wrong answer (minimum total score = 0, maximum total score = 11).

The other questionnaire used in the study was titled ‘Behavior questionnaire on prevention of CLABSI**’**. It was based on the questionnaire developed by Ullman et al. with some revisions [9]. The questionnaire consisted of closed-end questions investigating nurses' practices toward the prevention of CLABSIs. Answers were measured using a 5-point Likert-type scale, ranging from “never” to “always”.

### Internal consistency of questionnaire respondents

The Cronbach’s alpha of the ICU Nurses' Knowledge of Evidence-Based CLABSI Prevention questionnaire was 0.903, and of the behavior questionnaire on prevention of CLABSI was 0.924. These results indicate that the questionnaire results exhibited good internal consistency.

### Statistical analysis

No data was lost due to the use of online survey tools. Continuous variables were described as mean ± standard deviation (SD), or range (only for age). After evaluating the normality of the data distribution, the total scores were compared by the Student’s independent *t*-test between two groups. For comparison among three or more groups, the one-way ANOVA was conducted with the Bonferroni post-hoc pairwise comparisons. Categorical data were presented as number and percentage (%), and compared with the chi-square test or Fisher’s exact test (if any expected value ≤ 5 was found). The internal consistency of questionnaire respondents was assessed by Cronbach’s alpha, which is also a reliability index. The statistical significance level for all tests was set at a 2-tailed *P*-value < 0.05. All analyses were performed using IBM SPSS version 25 software (IBM Corporation, Somers, New York).

## Results

### Sample characteristics

The total number of visits to the questionnaires was 2607, and the number of nurses completing the questionnaires was 835. The overall questionnaire response rate was 835/2607 (32.03%). The characteristics of the 835 nurses who completed the questionnaires are summarized in Table 1. The mean age of all respondents was 26.54 years (range: 18–65 years). The nurses completing the questionnaires were from at least 104 hospitals, and 93% (777/835) of nurses were from hospitals in Guangdong Province. Workshops/courses were the primary method nurses obtained information on CLABSI prevention and 84.43% (705/835) of nurses indicated they needed more information about CLABSI.

**Table 1:** Respondent characteristics (N = 835)

Gender	
Female	498 (59.64%)
Male	337 (40.36%)
Age, years^a^	26.54 (18–65)
Length of ICU nursing, years	
< 1	435 (52.10%)
1– < 3	154 (18.44%)
3– < 5	99 (11.86%)
5–10	94 (11.26%)
> 10	53 (6.35%)
Highest educational level	
Secondary	117 (14.01%)
Tertiary school	337 (40.36%)
Undergraduate	335 (40.12%)
Postgraduate	46 (5.51%)
Nursing level	
Nurse	436 (52.22%)
Nurse practitioner	227 (27.19%)
Nurse-in-charge	118 (14.13%)
Deputy chief nurse and above	54 (6.47%)
Establishment of nurses	
Establishment nurses	301 (36.05%)
Personnel agent nurses	180 (21.56%)
Contract nurses	354 (42.40%)
Hospital grade	
Grade A tertiary hospital	553 (66.23%)
Grade B tertiary hospital	87 (10.42%)
Grade A secondary hospital	146 (17.49%)
Grade B secondary hospital	49 (5.87%)
Incidence of CLABSI in department last year (per 1000 catheter days)	
< 1‰	325 (38.92%)
1‰– < 3‰	232 (27.78%)
3‰–5‰	87 (10.42%)
> 5‰	20 (2.40%)
Do not know	171 (20.48%)
Sources of CLABSI information	
None	219 (26.23%)
Guidelines	304 (36.41%)
Workshops/courses	380 (45.51%)
Colleagues	282 (33.77%)
Scientific journals	201 (24.07%)
Internet	257 (30.78%)
Professional organization	220 (26.35%)
Do you feel you need more information about CLABSIs?	
No	130 (15.57%)
Yes	705 (84.43%)
Province	
Guangdong	777 (93.05%)
Other provinces	58 (6.95%)

CLABSI, central line-associated bloodstream infections; ICU, intensive care unit

^a^Age reported as mean and range

### ICU Nurses' Knowledge of CLABSI Prevention Questionnaire results

Table 2 describes the questions and results of the ‘Knowledge of CLABSI Prevention Questionnaire’. For the 11 individual questions, the percentage of correct responses ranged from 10.90 to 59.04%. Only one nurse answered all the questions in the whole questionnaire correctly. For all respondents, the overall correct response rate for the whole questionnaire was 36.56%. Only 16.29% (136/835) of nurses correctly indicated that CVCs should only be replaced if there is a specific indication. Only 10.90% (91/835) of nurses correctly answered that when there is continuous administration of liquids other than blood, blood products, or fat emulsions, the drug delivery device should be replaced every 96 h. Approximately 58% (485/835) of nurses correctly indicated that CVCs coated or impregnated with antimicrobial agents should be used in patients with an expected retention time of more than 5 days. Around 56% (467/835) of nurses correctly answered that when blood, blood products, or fat emulsions are given through a CVC, it is recommended that the drug delivery device be replaced every 24 h. Finally, about 60% (493/835) of nurses correctly indicated that when manipulating the catheter insertion site and hubs, it is recommended to use clean or sterile gloves and alcohol solutions/antiseptic soap.

**Table 2:** Responses to the ‘Knowledge of CLABSI Prevention Questionnaire’

Question number	Question	Number	Percentage of answers (%)
1	It is recommended to replace Central Venous Catheters (CVCs) routinely		
	(A) Yes, every 7 days	383	45.87
	(B) Yes, every 3 weeks	227	27.19
	(C) No, only when indicated*	136	16.29
	(D) I do not know	89	10.66
2	In settings with a high rate of catheter-related infections it is recommended to use a CVC coated or impregnated with an antiseptic agent		
	(A) Yes, in patients whose CVC is expected to remain in place for > 5 days*	485	58.08
	(B) No, because the use of such catheters is not cost-effective	102	12.22
	(C) No, because the use of such catheters does not result in a significant decrease in the rate of catheter-related infections	115	13.77
	(D) I do not know	133	15.93
3	It is recommended to change the dressing on the catheter insertion site…		
	(A) Every 2 days	257	30.78
	(B) Every 7 days	205	24.55
	(C) When indicated (e.g., soiled, loosened) and at least weekly*	280	33.53
	(D) I do not know	93	11.14
4	It is recommended to cover up the catheter insertion site with…		
	(A) Polyurethane dressing (transparent, semipermeable)	374	44.79
	(B) Gauze dressing	128	15.33
	(C) Both are recommended because they do not affect the risk for catheter-related infections*	237	28.38
	(D) I do not know	96	11.50
5	It is recommended to disinfect the catheter insertion site with…		
	(A) 70% alcohol	275	32.93
	(B) 2% chlorhexidine gluconate with alcohol*	328	39.28
	(C) Povidone-iodine	118	14.13
	(D) I do not know	114	13.65
6	It is recommended to apply an antibiotic ointment at the insertion site of CVC		
	(A) Yes, because it decreases the risk for catheter-related infections	356	42.63
	(B) No, because it causes antibiotic resistance*	178	21.32
	(C) No, because it does not decrease the risk for catheter-related infections	153	18.32
	(D) I do not know	148	17.72
7	When blood, blood products, or lipid emulsions are administered through a CVC, it is recommended to replace the administration set…		
	(A) Within 24 h*	467	55.93
	(B) Every 72 h	184	22.04
	(C) Every 96 h	69	8.26
	(D) I do not know	115	13.77
8	When liquids other than blood, blood products, or fat emulsions are administered continuously the administration set should be replaced		
	(A) Every 24 h	448	53.65
	(B) Every 48 h	183	21.92
	(C) Every 96 h*	91	10.90
	(D) I do not know	113	13.53
9	It is recommended to use an antiseptic agent to clean the access hub or connector before the connection of the administration set or after unscrewing the dead-end cap closes the catheter		
	(A) Yes, by spraying the access site with 70% alcohol solution or alcohol chlorhexidine solution	285	34.13
	(B) Yes, by wiping with 70% alcohol solution or alcohol and chlorhexidine solution for no less than 15 s*	309	37.01
	(C) It is not recommended because no evidence has been found for the relation between the disinfections of the connecting site of the administration set and the contamination of fluids or the insertion hub	124	14.85
	(D) I do not know	117	14.01
10	When manipulating the catheter insertion site and hubs, it is recommended…		
	(A) To obviate hand hygiene if gloves are used and water for hand hygiene before manipulation	203	24.31
	(B) To use clean or sterile gloves and alcohol solutions/antiseptic soap*	493	59.04
	(C) Hand hygiene is only necessary before catheter insertion	36	4.31
	(D) I do not know	103	12.34
11	It is recommended to replace pressure transducers and tubing routinely…		
	(A) Yes, every 4 days*	354	42.40
	(B) Yes, every 7 days	280	33.53
	(C) No, only when indicated	90	10.78
	(D) I do not know	111	13.29
	Total correct answers (%)		36.56

^*^Indicates the correct answer

### Subgroup analyses of ‘ICU Nurses' Knowledge of Evidence-Based CLABSI Prevention’ results stratified by demographic and other variables

**Fig. 1:**
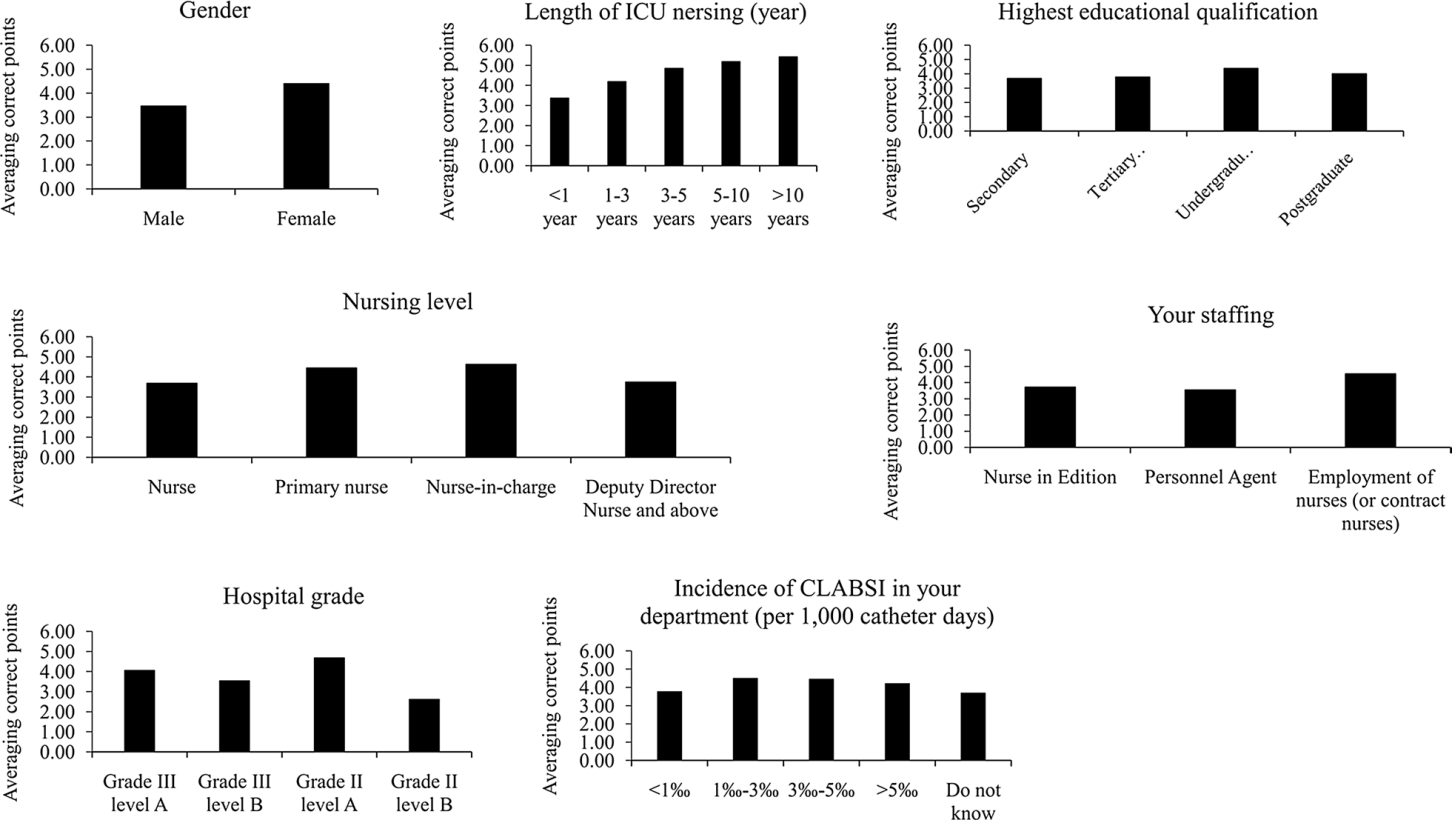
Bar charts of subgroup analyses of mean correct scores stratified by sex, length of ICU nursing, highest educational level, nursing level, staffing, hospital grade, and incidence of CLABSIs in respondent’s department in the past year (per 1000 catheter days)

Results of the Subgroup analyses stratified by demographic and other variables are summarized in Table 3 and Fig. 1. The mean overall score for the 11 questions was 4.02. It was found that the total knowledge score was significantly different in all the subgroup analyses stratified by Sex, length of time of ICU nursing, educational level, nursing level,establishment of nurses,hospital grade, and incidence of CLABSI. Female nurses had significantly higher scores than male nurses (*P* < 0.001), and experienced nurses had higher scores than less experienced nurses (*P* < 0.001). There was significant difference among the groups of different length of ICU nursing (*P* = 0.001). A trend could be found that the longer a nurse worked in the ICU, the higher the questionnaire score. Nurses who graduated from undergraduate courses had significantly higher scores than those who graduated from junior and technical secondary schools (*P* < 0.001). Nursing level also affected questionnaire score: the scores of nurses were significantly lower than those of nurse practitioner and nurse-in-charge (*P* < 0.001).

**Table 3:** Comparison of ‘Knowledge of CLABSI Prevention Questionnaire’ based on demographic and other variables

Characteristics	Mean ± SD	Range	Significantly different from group	t/F	*P*
Total cohort (N = 835)	4.02 ± 2.11	0–11			
Sex				6.53	< 0.001
Male (n = 337)	3.46 ± 1.92	0–9	Female		
Female (n = 498)	4.40 ± 2.15	0–11	Male		
Length of ICU nursing				31.89	< 0.001
A. < 1 year (n = 435)	3.36 ± 1.91	0–9	B, C, D, E		
B. 1– < 3 years (n = 154)	4.18 ± 1.82	0–8	A, D, E		
C. 3– < 5 years (n = 99)	4.85 ± 2.15	0–9	A		
D. 5–10 years (n = 94)	5.18 ± 2.03	0–10	A, B		
E. > 10 years (n = 53)	5.42 ± 2.35	0–11	A, B		
Highest educational level				5.92	0.001
A. Secondary school (n = 117)	3.68 ± 1.88	0–9	C		
B. Tertiary school (n = 337)	3.78 ± 2.08	0–9	C		
C. Undergraduate (n = 335)	4.39 ± 2.12	0–10	A, B		
D. Postgraduate (n = 46)	4.00 ± 2.40	0–11	–		
Nursing level				10.75	< 0.001
A. Nurse (n = 436)	3.68 ± 1.98	0–9	B, C		
B. Nurse practitioner (n = 227)	4.44 ± 2.14	0–10	A		
C. Nurse-in-charge (chief nurse) (n = 118)	4.63 ± 2.23	0–11	A		
D. Deputy chief nurse and above (n = 54)	3.74 ± 2.11	0–9	–		
Establishment of nurses				18.89	< 0.001
A. Establishment nurses (n = 301)	3.71 ± 1.93	0–11	C		
B. Personnel agent nurses(n = 180)	3.54 ± 1.93	0–9	C		
C. Contract nurses (n = 354)	4.53 ± 2.23	0–10	A, B		
Hospital grade				14.20	< 0.001
A. Grade A tertiary hospital (n = 553)	4.05 ± 1.95	0–11	C, D		
B. Grade B tertiary hospital (n = 87)	3.53 ± 2.05	0–8	C		
C. Grade A secondary hospital (n = 146)	4.67 ± 2.38	0–10	A, B, D		
D. Grade B secondary hospital (n = 49)	2.61 ± 2.23	0–8	A, C		
Incidence of CLABSI in department last year (per 1000 catheter days)				6.19	< 0.001
A. < 1‰ (n = 325)	3.75 ± 1.93	0–9	B		
B. 1‰– < 3‰ (n = 232)	4.48 ± 1.94	0–10	A, E		
C. 3‰–5‰ (n = 87)	4.44 ± 1.97	0–9	–		
D. > 5‰ (n = 20)	4.20 ± 1.85	2–9	–		
E. Do not know (n = 171)	3.68 ± 2.57	0–11	B		

CLABSI, central line-associated bloodstream infection

Post-hoc comparisons were performed using the Bonferroni test when overall significance was indicated by one-way ANOVA

In China, nurses can be divided into 3 categories: establishment nurses, personnel agent nurses, and contract nurses according to whether they have national establishment or not. Contract nurses scored significantly higher than establishment nurses and personnel agent nurses (*P* < 0.001). The incidence of CLABSI in the ICU was also associated with questionnaire score: nurses in ICUs with an incidence of 1–3‰ were significantly higher than nurses in ICUs with an incidence of < 1‰ and higher than that of nurses who answered “I don't know” (both, *P* < 0.05).

### Results of the Behavior questionnaire on prevention of CLABSI

Results of the Behavior questionnaire on prevention of CLABSI of ICUs in China are summarized in Table 4. Approximately 43% (363/835) of nurses reported always using maximum barrier precautions, while around 15% (121/835) reported never using 2% chlorhexidine gluconate. Around 39% (323/835) of nurses reported replacing administration sets at least every 7 days, but no more frequently than at 96-h intervals. Around 33% (273/835) of nurses reported frequently and routinely changing catheters, even if there was no suspicion of a CLABSI.

**Table 4:** Results of the ‘Behavior questionnaire on prevention of CLABSI**’**

How frequently are these practices used in your facility?	Never, n (%)	Rarely, n (%)	Sometimes, n (%)	Mostly, n (%)	Always, n (%)
Maximum barrier precautions (cap, mask, sterile gown, sterile gloves, and a sterile full body drape)	110 (13.17%)	123 (14.73%)	102 (12.22%)	137 (16.41%)	363 (43.47%)
2% Chlorhexidine gluconate for antisepsis of the insertion site	121 (14.49%)	122 (14.61%)	151 (18.08%)	132 (15.81%)	309 (37.01%)
Use of suture-less securement devices	126 (15.09%)	163 (19.52%)	196 (23.47%)	131 (15.69%)	219 (26.23%)
Use of sterile, transparent, semi-permeable dressing to cover catheter site	86 (10.30%)	103 (12.34%)	170 (20.36%)	134 (16.05%)	342 (40.96%)
Transparent dressing replaced at least every 7 days	106 (12.69%)	103 (12.34%)	141 (16.89%)	142 (17.01%)	343 (41.08%)
Administration sets replaced no more frequently than at 96-h intervals, but at least every 7 days	107 (12.81%)	113 (13.53%)	149 (17.84%)	143 (17.13%)	323 (38.68%)
Prompt removal of catheter when no longer essential	87 (10.42%)	102 (12.22%)	175 (20.96%)	136 (16.29%)	335 (40.12%)
Routine catheter changes even if there is no suspicion of a CLABSI	115 (13.77%)	123 (14.73%)	192 (22.99%)	132 (15.81%)	273 (32.69%)

## Discussion

The questionnaire surveys used in this study indicate that there is a fundamental gap between knowledge, and practice in the care of CVC and prevention of CLABSIs by ICU nurses in China. The participants in this study were ICU nurses from at least 104 hospitals in China. Although guidelines have been published for preventing CLABSIs, including guidelines published in China [5, 18–20], our results indicated that Chinese ICU nurses have only a modest understanding of the basic principles needed to prevent CLABSIs. The findings are similar to those reported in some other countries where health care workers generally have low understanding of CLABSI prevention guidelines [9, 11, 15, 21–23].

Since the care of the catheter insertion site is usually a nursing responsibility, it would be expected that nurses would overall answer questions on this topic correctly. However, our results did not find this assumption true. Only 16% of nurses correctly answered that CVCs did not need to be replaced routinely at a set interval. Only approximately 11% (91/835) of nurses correctly answered that when continuous administration of liquids other than blood, blood products, or fat emulsions is performed, the drug delivery device should be replaced every 96 h. The rates of correct answers to these 2 questions were significantly lower than in other countries [9, 11, 15, 22, 23]. Furthermore, the overall correct answer rate of the ICU Nurses' Knowledge of Evidence-Based CLABSI Prevention Questionnaire was only 37%, which is also lower than the result (44.4%) of a knowledge test among 3405 European ICU nurses [11]. This indicates that a great deal of improvement of ICU nurses’ knowledge of evidence-based guidelines for the prevention of CLASBSIs is needed in China.

Our results also indicated that sex, length of time of ICU nursing, educational level, nursing level, establishment of nurses, hospital grade, and incidence of CLABSI were significantly associated with total knowledge score of the ICU Nurses' Knowledge of Evidence-Based CLABSI Prevention Questionnaire. These results are similar to those of other studies [11, 15, 22], and suggest that more years practicing nursing, which leads to greater experience, and formalized education can improve knowledge and practice of evidence-based guidelines for preventing CLABSIs.

In the study, 59.02% (435/835) nurses worked in an ICU for < 1 year. Thus, the proportion of nurses with a relatively low ICU experience level was large. One reason for this is that the students who helped distribute the questionnaire had graduated in 2019 and thus nurses most accessible to them were those who had recently graduated as well and thus did not have many years of experience. This is a selection bias caused by RDS. Nurses who had worked in an ICU for 3 or more years had a correct answer rate of 45.45% (5/11) of the questionnaire, which is similar to the results of other studies [9, 11].

In China, for nurses performing the same duties, establishment nurses are the highest paid, followed by personnel agent nurses. However, "equal pay for equal work" is being advocated in China, and the pay gap between different classes of nurses is getting smaller [24]. Our results showed that contract nurses scored higher on the prevention knowledge of CLABSI than the other two categories of nurses. Further research is needed to determine the causes of this result.

Our results also showed that in addition to a lack of knowledge of evidence-based guidelines to prevent CLABSIs by the CDC, US and the Department of Health UK, the actual practice of the techniques and methods to prevent infections was poor as compared to the results of other studies examining practices in ICUs [9, 22]. This includes the use of 2% chlorhexidine gluconate, replacement groups, maximum aseptic barrier precautions, application of transparent dressings, and removal of catheters, the application of suture-free fixation instruments. On-the-job training is needed to promote the application of evidence-based nursing measures for central venous catheters in China.

There are still some limitations to this study. The study provides a comprehensive examination of ICU nursing knowledge and practices in Guangdong Province. However, the results may not reflect the knowledge and practices of ICU nurses in other parts of China. In addition, the overall questionnaire response rate was 835/2607 (32.03%), which can also lead to bias. Half of the respondents worked in an ICU for < 1 year, which contributed to the overall low scores. Our results did show that nurses with more experience had higher scores. A marked strength of this study is the large number of participants and the large number of hospitals represented; thus, the findings can be considered an accurate representation of the geographic region.

## Conclusion

The results of this study indicate that Chinese ICU nurses in Guangdong Province have a great lack of knowledge and practice of evidence-based guidelines for the prevention of CLABSIs. These results suggest that national health administrations should adopt policies to provide training for ICU nurses regarding the evidence-based guidelines and practices to prevent CLABSIs.

## Data Availability

All the data and material were presented in the main paper.

## Abbreviations

CLABSIs: Central line-associated bloodstream infections
CVCs: Central venous catheters
ICUs: Intensive care units
MHA: Michigan Health and Hospital Association
SD: Standard deviation

## References

[1] Rosenthal VD, Al-Abdely HM, El-Kholy AA, AlKhawaja SAA, Leblebicioglu H, Mehta Y (2016). International Nosocomial Infection Control Consortium report, data summary of 50 countries for 2010–2015: device-associated module. Am J Infect Control.

[2] Kusek L (2012). Preventing central line-associated bloodstream infections. J Nurs Care Qual.

[3] Ziegler MJ, Pellegrini DC, Safdar N (2014). Attributable mortality of central line associated bloodstream infection: systematic review and meta-analysis. Infection.

[4] Haddadin Y, Annamaraju P, Regunath H. Central Line Associated Blood Stream Infections (CLABSI). StatPearls. StatPearls Publishing; 2020. 28613641

[5] Pronovost P, Needham D, Berenholtz S, Sinopoli D, Chu H, Cosgrove S (2006). An intervention to decrease catheter-related bloodstream infections in the ICU. N Engl J Med.

[6] Ling ML, Apisarnthanarak A, Jaggi N, Harrington G, Morikane K, Thu LTA (2016). APSIC guide for prevention of Central Line Associated Bloodstream Infections (CLABSI). Antimicrob Resist Infect Control.

[7] Zeng C, Chen Y, Jia H, Li L, Wu A (2014). Investigation of catheter-related bloodstream infections in ICU, Xiangya Hospital CSU. Chin J Nosocomiol.

[8] Perin DC, Erdmann AL, Higashi GDC, Dal Sasso GTM (2016). Evidências de cuidado para prevenção de infecção de corrente sanguínea relacionada a cateter venoso central: Revisão sistemática. Rev Lat Am Enfermagem.

[9] Ullman AJ, Long DA, Rickard CM (2014). Prevention of central venous catheter infections: a survey of paediatric ICU nurses’ knowledge and practice. Nurse Educ Today.

[10] Bianco A, Coscarelli P, Nobile CGA, Pileggi C, Pavia M (2013). The reduction of risk in central line-associated bloodstream infections: Knowledge, attitudes, and evidence-based practices in health care workers. Am J Infect Control.

[11] Labeau SO, Vandijck DM, Rello J, Adam S, Rosa A, Wenisch C (2009). Centers for Disease Control and Prevention guidelines for preventing central venous catheter-related infection: results of a knowledge test among 3405 European intensive care nurses. Crit Care Med.

[12] Koutzavekiaris I, Vouloumanou EK, Gourni M, Rafailidis PI, Michalopoulos A, Falagas ME (2011). Knowledge and practices regarding prevention of infections associated with central venous catheters: a survey of intensive care unit medical and nursing staff. Am J Infect Control.

[13] Esposito MR, Guillari A, Angelillo IF (2017). Knowledge, attitudes, and practice on the prevention of central line-associated bloodstream infections among nurses in oncological care: a cross-sectional study in an area of southern Italy. PLoS ONE.

[14] Liu YJ, Han N, Cai PLS (2012). Reliability and validity analysis of cognitive and behavioral questionnaires for venous catheter-related infection in the prevention center. Chin J Nurs.

[15] Dedunska KDD (2015). No Prevention of central venous catheter-associated bloodstream infections: a questionnaire evaluating the knowledge of the selected 11 evidence-based guidelines by Polish nurses. Am J Infect Control.

[16] O’Grady NP, Alexander M, Dellinger EP, Gerberding JL, Heard SO, Maki DG (2002). Guidelines for the prevention of intravascular catheter-related infections. Am J Infect Control.

[17] Loveday HP, Wilson JA, Pratt RJ, Golsorkhi M, Tingle A, Bak A (2014). Epic3: National evidence-based guidelines for preventing healthcare-associated infections in nhs hospitals in england. J Hosp Infect.

[18] O’Grady NP, Alexander M, Burns LA, Dellinger EP, Garland J, Heard SO (2011). Guidelines for the prevention of intravascular catheter-related infections. Am J Infect Control.

[19] Marschall J, Mermel LA, Fakih M, Hadaway L, Kallen A, O’Grady NP (2014). Strategies to prevent central line-associated bloodstream infections in acute care hospitals: 2014 update. Infect Control Hosp Epidemiol.

[20] FQ (2008). Guidelines for the prevention and treatment of endovascular catheter-related infections. Chin J Pract Surg.

[21] Al QM (2017). Oncology nurses’ knowledge of guidelines for preventing catheter-related bloodstream infections. Am J Infect Control.

[22] Guembe M, Bustinza A, Sánchez Luna M, Carrillo-álvarez A, Pérez Sheriff V, Bouza E (2012). Guidelines for preventing catheter infection: assessment of knowledge and practice among paediatric and neonatal intensive care healthcare workers. J Hosp Infect.

[23] Guembe M, Pérez-Parra A, Gómez E, Sánchez-Luna M, Bustinza A, Zamora E (2012). Impact on knowledge and practice of an intervention to control catheter infection in the ICU. Eur J Clin Microbiol Infect Dis.

[24] Huigen H, Fu X, Xu JJCS (2011). Comparison of the quality of life between contract nurses and nurses in the tertiary hospital in Guangdong Province. J Nurs.

